# Changes in microparticle profiles by vitamin D receptor activation in chronic kidney disease – a randomized trial

**DOI:** 10.1186/s12882-019-1445-4

**Published:** 2019-08-01

**Authors:** Kristina Lundwall, Josefin Mörtberg, Fariborz Mobarrez, Stefan H. Jacobson, Gun Jörneskog, Jonas Spaak

**Affiliations:** 1 Department of Cardiology, Danderyd University Hospital, 182 88 Stockholm, Sweden; 2 Department of Nephrology, Danderyd University Hospital, 182 88 Stockholm, Sweden; 3 Department of Medicine, Danderyd University Hospital, 182 88 Stockholm, Sweden; 4 Department of Clinical Sciences, Danderyd University Hospital, Karolinska Institutet, Stockholm, Sweden

**Keywords:** Microparticles, ICAM-1, Endothelial function, Vitamin D, Chronic kidney disease

## Abstract

**Background:**

Microparticles (MPs) are biomarkers and mediators of disease through their expression of surface receptors, reflecting activation or stress in their parent cells. Endothelial markers, ICAM-1 and VCAM-1, are implicated in atherosclerosis and associated with cardiovascular risk. Chronic kidney disease (CKD) patients have endothelial dysfunction and high levels of endothelial derived MPs. Vitamin D treatment has been reported to ameliorate endothelial function in CKD patients. We aimed to examine cell specific MP profiles and concentrations of MPs expressing the atherosclerotic markers ICAM-1 and VCAM-1 after treatment with paricalcitol in patients with CKD stage 3–4.

**Methods:**

Sub-study of the previously reported SOLID trial where 36 patients were randomly assigned to placebo, 1 or 2 μg paricalcitol, for 12 weeks. MPs were measured by flow cytometry after labelling with antibodies against endothelial (CD62E), platelet (CD62P, CD41, CD154) leukocyte (CD45) and vascular (CD54, CD106) markers.

**Results:**

Patients had a mean age of 65 years with a mean eGFR of 40 mL/min/1.73m^2^. Concentrations of ICAM-1 positive MPs were significantly reduced by treatment (repeated measures ANOVA *p* = 0.04). Repeated measures MANOVA of concentrations of endothelial, platelet and leukocyte MPs showed sustained levels in the 2 μg treatment group (*p* = 0.85) but a decline in the 1 μg (*p* = 0.04) and placebo groups (*p* = 0.005).

**Conclusions:**

Treatment with paricalcitol reduces concentrations of ICAM-1 positive MPs. This is accompanied by sustained concentrations of all cell specific MPs in the 2 μg group, and decreasing concentrations in the other groups, possibly due to a more healthy and reactive endothelium with paricalcitol treatment.

## Background

The importance of the close association between chronic kidney disease (CKD) and cardiovascular disease (CVD), the cardiorenal syndrome, is becoming increasingly recognised among nephrologists and cardiologists [1–3]. The mechanisms underlying this association have been explored during the last decade, identifying an accelerated vascular disease with early endothelial dysfunction [4], and subsequent vascular calcification progressing to arterial stiffening and remodelling [3, 5–8].

Vitamin D is currently regarded as a hormone with multiple pleiotropic effects, and the deficiency of the activated form in CKD has attracted substantial attention [9–12]. Vitamin D seems to have endothelial protective properties, downregulating both oxidative stress and the inflammatory response, and upregulating the key enzyme for endothelial function, eNOS [13–17]. Treatment or supplementation with vitamin D compounds to CKD patients seems to lower albuminuria [18–20]. Regarding measures of vascular function, several studies and a recent meta-analysis by our group have shown positive effects on endothelial function [10, 21–24], although this might be debated in lower doses [25, 26]. Regarding measures of arterial stiffness, the next step in the vascular disease process, most [27, 28] but not all [10, 11] studies have been negative.

Microparticles are small vesicles shedded from the parent cell membrane as a response to activation of the cell, but also during stress and apoptosis [29–31]. Evidence is emerging showing that MPs are biologically active, affecting cells locally and mediating intercellular communication [29, 30, 32–34]. The mechanism for their release, apoptosis, cell damage or activation, seems to induce different patterns of markers such as CD31, CD62E, CD62P, or CD144 on their surface [29, 31, 35]. Depending on their origin and surface molecules, they exhibit opposite effects on apoptosis, inflammation and oxidative stress [29, 31–33, 36, 37].

Levels of CD31-positive-CD41-negative (CD31^+^ CD41^**−**^) and CD144-positive (CD144^+^) endothelial MPs (EMPs) correlate with physiological measurements of vascular function, such as flow mediated vasodilatation (FMD) and pulse wave velocity (PWV) [38–42]. The marked endothelial dysfunction in CKD patients is consequently reflected in higher levels of CD144^+^ and CD31^+^CD41^−^EMPs compared to healthy controls [38, 43–45]. For CD144^+^, and CD31^+^CD41^−^ EMPs, elevated levels are associated with cardiovascular morbidity and mortality in atherosclerosis, kidney failure, pulmonary hypertension and heart failure [39, 46–49].

Intercellular adhesion molecule 1 (ICAM-1; CD54) and vascular cell adhesion molecule 1 (VCAM-1; CD106) are expressed on endothelial cells and on EMPs from activated and proinflammatory cells [31, 35]. They control the recruitment and migration of white blood cells to the site of inflammation and play an important role in the atherosclerotic process [50]. Some studies indicate that levels of soluble ICAM-1 and VCAM-1 correlate with cardiovascular events and death in CKD patients [51] [52, 53], to intima media thickness in hemodialysis (HD) patients [54], and to measures of dyslipidaemia [52, 53]. ICAM-1 and VCAM-1 are therefore considered to reflect the level of endothelial proinflammatory and proatherosclerotic activation in the endothelium [50].

This is a sub-study of the SOLID trial in which we show that active vitamin D attenuates a decline in endothelial function measured by FMD, and reduces levels of proinflammatory cytokines [15, 22]. The effects of vitamin D treatment on MPs and their expression of surface proteins in CKD patients have until now not been studied. We therefore hypothesized that these protective vascular effects of vitamin D treatment would be reflected in the concentrations of ICAM-1 and VCAM-1 positive MPs, and potentially also in the concentrations of CD62E^+^ EMPs, as well as other cellspecific MPs.

## Methods

### Participants

This is a sub-study of the blood samples collected in the SOLID trial [22]. Department of Nephrology at Danderyd University Hospital, Stockholm, Sweden, was the base of recruitment, between June 2010 and February 2013. Inclusion criteria were an estimated glomerular filtration rate (eGFR) of 15–59 mL/min/1.73m^2^ (plasma creatinine used in the Modification of Diet in Renal Disease (MDRD) formula), age > 20 years, a calcium level below 2.6 mmol/L, a plasma PTH level of 3.7–53 pmol/L, a serum albumin above 30 g/L and with no changes in angiotensin converting enzyme inhibitor or angiotensin receptor blocker 2 months before the trial. Most important exclusion criteria were diabetes mellitus or treatment with vitamin D or its analogues, acute renal failure during the last 3 months, uncontrolled hypertension (repeated measures of a brachial blood pressure > 150/100 mmHg), or other severe disease (severe congestive heart failure, active cancer or AIDS/HIV). The study protocol was approved by the regional Ethics Committee of Stockholm, Sweden. All patients provided written informed consent. The trial was registered on clinicaltrials.gov (SOLID study; NCT01204528) in April 27, 2010.

### Study design and procedures

**Fig. 1:**
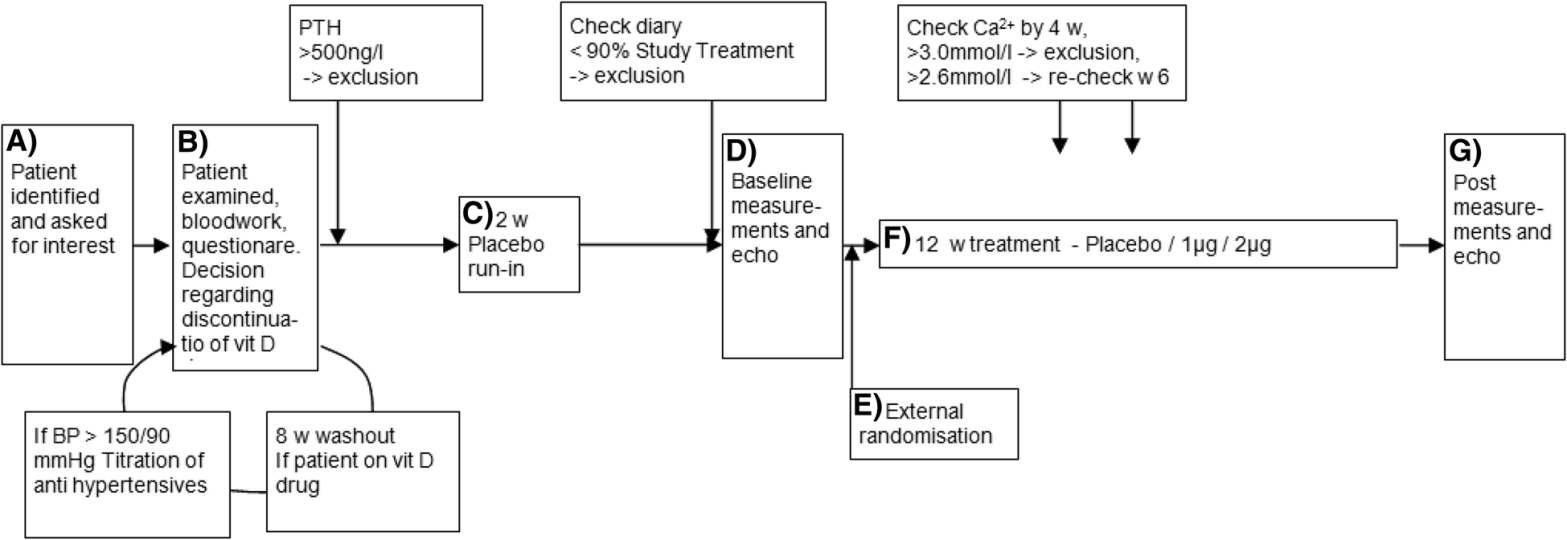
Flow diagram of the SOLID trial

The complete study design and procedures have already been published [22] (see Fig. 1, flow diagram of the solid trial). Patients were included and randomized consecutively and double blinded, to three groups; placebo, 1 μg paricalcitol, or 2 μg paricalcitol daily. Patients were started on 2 weeks of placebo run in, followed by 12 weeks of blinded treatment. Measurements including blood samples were made at baseline and after intervention. Patients came in the morning, fasting (12 h) and rested for 20 min. Venous blood samples were then drawn, for routine chemistry in the hospital laboratory, for centrifugation to platelet poor plasma, and then frozen for later specialized assays, among them MP measurements. MP measurements were performed in research laboratories at Danderyd University Hospital, Stockholm, Sweden. To avoid the influence of sun exposure and vitamin D activation, no patients were randomized during June to August.

### MP measurements by flow cytometry

Frozen (− 80 degrees Celcius) platelet poor plasma (PPP) were thawed in water bath and centrifuged initially at 2,000 g for 20 min at room temperature The supernatant was then centrifuged again at 13,000 g for 2 min at room temperature. Subsequently, 20 μL of the supernatant was incubated in the dark with 5 μl lactadherin-FITC (80 μM, BLAC-FITC, Coatech AB, Klädesholmen, Sweden) that binds to phosphatidylserine (PS), together with 5 μl CD62E-APC (0.25 μg/test, Thermo Fisher Scientific, Waltham, MA, USA), CD41-APC (5 μl, Beckman Coulter, Brea, CA, USA), 5 μl CD62P-PE (0.25 μg/test, Thermo Fisher Scientific, Waltham, MA, USA), 5 μl CD154-APC (0.25 μg/test, Thermo Fisher Scientific, Waltham, MA, USA) and 5 μl CD45-APC (Beckman Coulter, Brea, CA, USA). In addition, the concentrations of 5 μl CD106-PE positive MPs (VCAM-1, Abcam, Cambridge, UK) and 5 μl CD54-PE positive MPs (0.25 μg/ml ICAM-1, Abcam Cambridge, UK) were investigated. ICAM-1 and VCAM-1 were considered as primary analysis, since they are more validated as risk markers. MP profile was considered a secondary analysis.

**Fig. 2:**
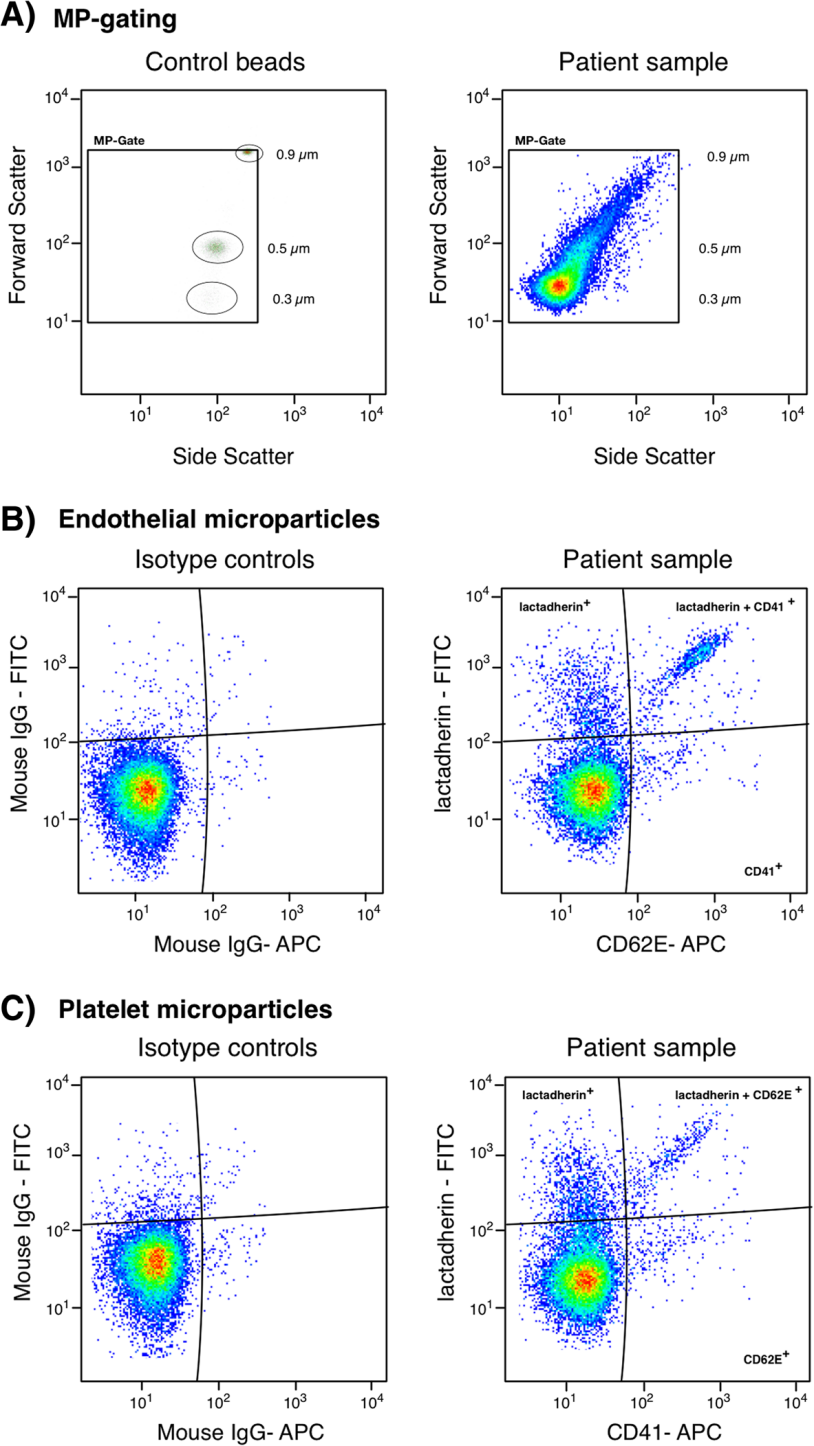
Microparticle gating strategy. Representative flow cytometric plots demonstrating **a**) MP-gate alignment by beads and a patient sample. **b** Endothelial microparticles labeled with isotope controls and lactadherin-FITC and CD62E-APC. **c** Platelet microparticles labeled with isotope controls and lactadherin-FITC and CD41-APC. All samples were measured on a Gallios flow cytometer

All samples were measured on a Gallios flow cytometer (Beckman Coulter, Brea, CA, USA). The MP gate was determined with Megamix beads (FSC; 0.3 μm, 0.5 μm, 0.9 μm BioCytex, Marseille, FR) and MPs were defined as particles less than 0.9 μm. MPs were categorized by size and lactadherin binding together with cell-specific antibodies, or only size and exposure of CD106 or CD54. Conjugate isotype-matched immunoglobulin (Mouse IgG, FITC, PE, APC; all from Beckman Coulter) with no reactivity against human antigens was used as a negative control to define the background noise of the cytometric analysis. The MP counts are presented as MPs per μL plasma (see Fig. 2 for gating strategy).

### Statistical analyses

All analyses were made according to intention to treat. Equality of variances in the sample was tested by Levene’s Test of Equality of Error Variances, and the assumptions for ANOVA were fulfilled for all parameters. Baseline comparisons were performed with oneway ANOVA, with Pearson’s chi-squared, and with Fischer’s exact test when appropriate. To investigate the effect of treatment on cell-specific MP subtypes, while avoiding multiple comparisons, we used repeated measures two-way MANOVA with time and treatment as independent variables. All MPs with cell specific antibody labelling were included as dependent variables. Post hoc analysis for within group changes was made by repeated measures one way (split by treatment group) MANOVA and in a separate analysis of EMPs by paired t-test. Effects of treatment on the concentrations of ICAM-1 and VCAM-1 positive microparticles were investigated with repeated measures two-way ANOVA. Since our study was the first on the subject, and a sub-study of the SOLID trial, no power calculations were made, and it was thus considered as hypothesis generating. A *p*-value < 0.05 was considered significant. All analyses were made with SPSS version 24 (IBM Corp. 2016).

## Results

### Patient characteristics

Thirty-six patients were included and randomized to placebo, 1 or 2 μg of paricalcitol. One patient experienced side effects of treatment (placebo) and therefore did not continue on intervention and declined to do the post measurements. No major adverse events were recorded during the study.

Baseline characteristics are presented in Table 1. Included patients were well matched in terms of kidney function and treatment. Neither was there any difference in baseline plasma levels of 25OH-vitamin D, calcium, phosphate or levels of parathyroid hormone (PTH). They were also well matched in vascular parameters such as FMD, PWV, iontophoresis and echocardiographic measures, previously reported in detail [22]. The placebo group was however slightly older than the 2 μg group (*p* = 0.02). Correlations at baseline were performed, showing no correlation between age and outcome parameters, as well as age and vascular parameters. There was, in absolute numbers, more CV disease in the placebo group, compared to the 2 μg group, but this difference was not statistically significant.

**Table 1:**
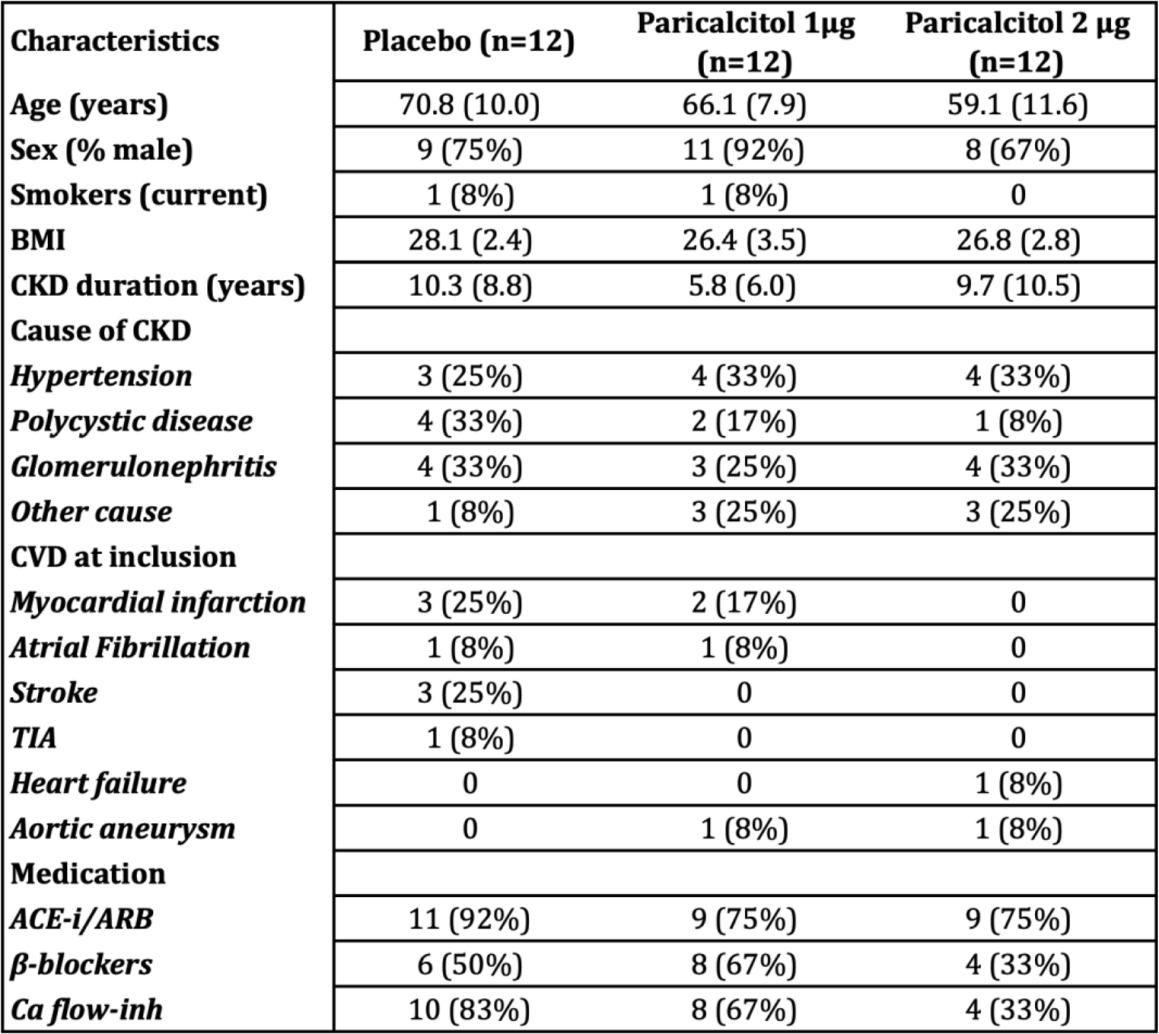
Baseline characteristics

*BMI* Body mass index, *CKD* Chronic kidney disease, *CVD* Cardiovascular disease, *TIA* Transient ischaemic attack, *ACE-i* Angiotensin converting enzyme inhibitor, *ARB* Angiotensin receptor blocker, *Ca flow-inh* Calcium flow inhibitor. Values are expressed as mean (SD)

### Blood chemistry post treatment

Results are presented in Table 2. PTH was suppressed by treatment (ANOVA *p* = 0.006) in a dose dependent manner. There was no significant change in phosphate or calcium levels by treatment. However, we saw slightly higher calcium levels in absolute numbers in the 2 μg group. Levels of FGF-23 were not significantly changed by treatment. Estimated GFR was not affected by treatment, but there was numerically a non significant decline in renal function in all groups during the study (repeated measures ANOVA main effect of time *p* = 0.06).

**Table 2:**
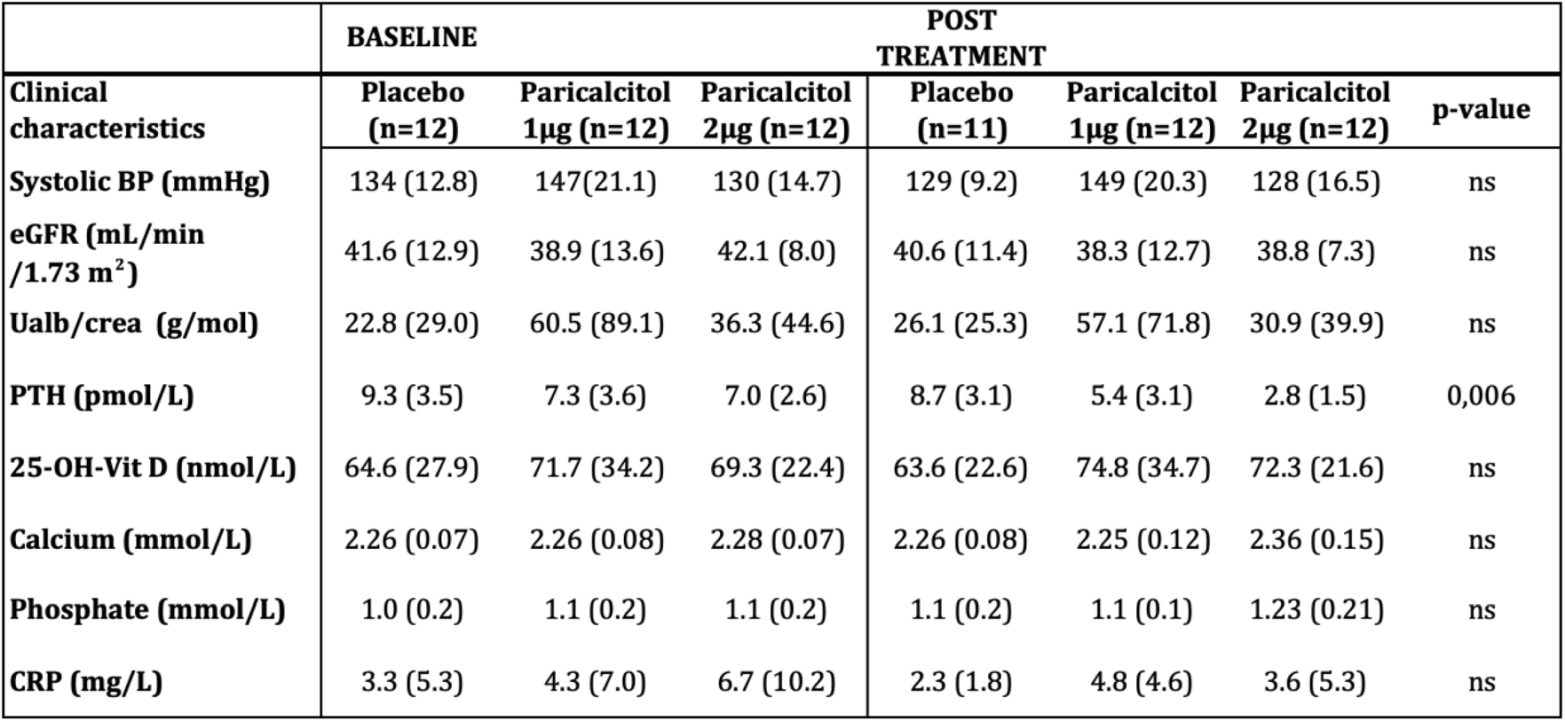
Clinical characteristics and laboratory measurements pre- and post-treatment

*BP* Blood pressure, *eGFR* Estimated glomerular filtration rate, Ualb/crea = urine albumin creatinine ratio, *PTH* Parathyroid hormone, *FGF23* Fibroblast growth factor 23. Values are expressed as mean (SD). *P*-value for interaction of treatment and time

### ICAM-1 and VCAM-1 positive microparticles

**Fig. 3:**
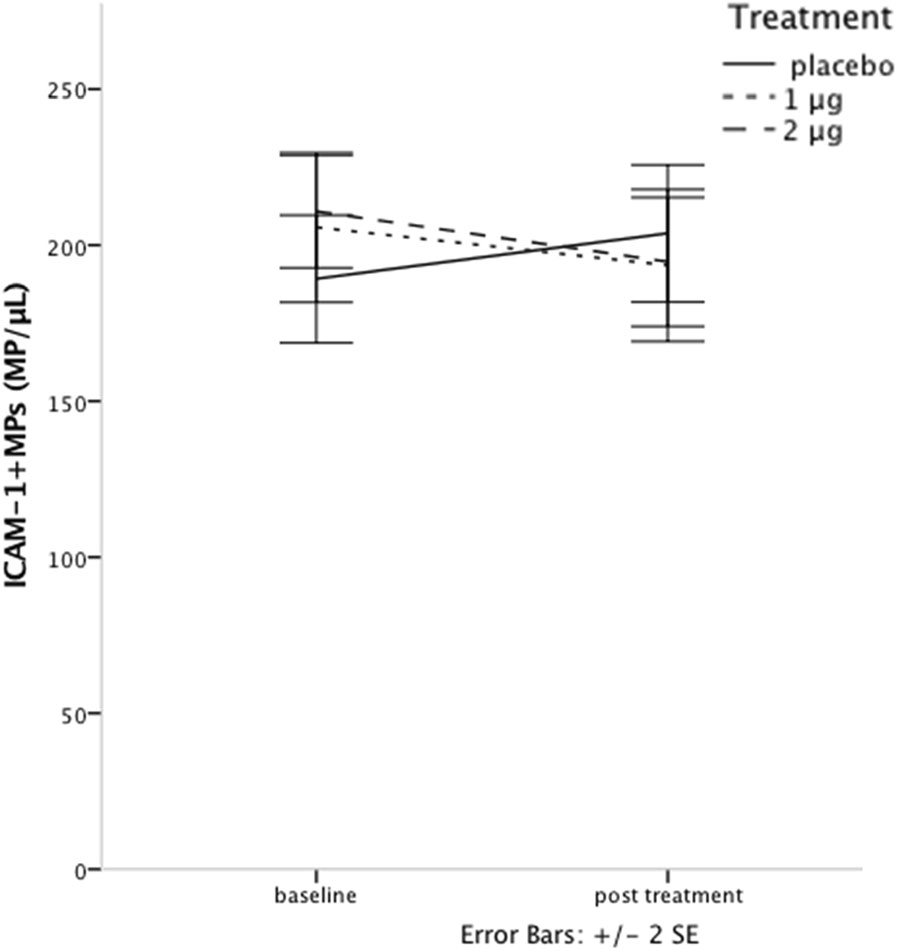
ICAM-1^+^ MP concentrations at baseline and post-treatment show significant interaction between treatment and time, i.e. the change over time was different between the placebo group and treated groups

There were no significant between-group differences in concentrations of ICAM-1^+^ or VCAM-1^+^ MPs before treatment. Concentrations of ICAM-1^+^ MPs demonstrated a significant interaction between time and treatment (repeated measures ANOVA *p* = 0.04), with a rise in MPs expressing ICAM-1 in the placebo group and a decline in the treated groups (Fig. 3, Table 3). Concentrations of VCAM-1^+^ MPs did not change significantly during the study (repeated measures ANOVA *p* = 0.52) (Table 3).

**Table 3:**
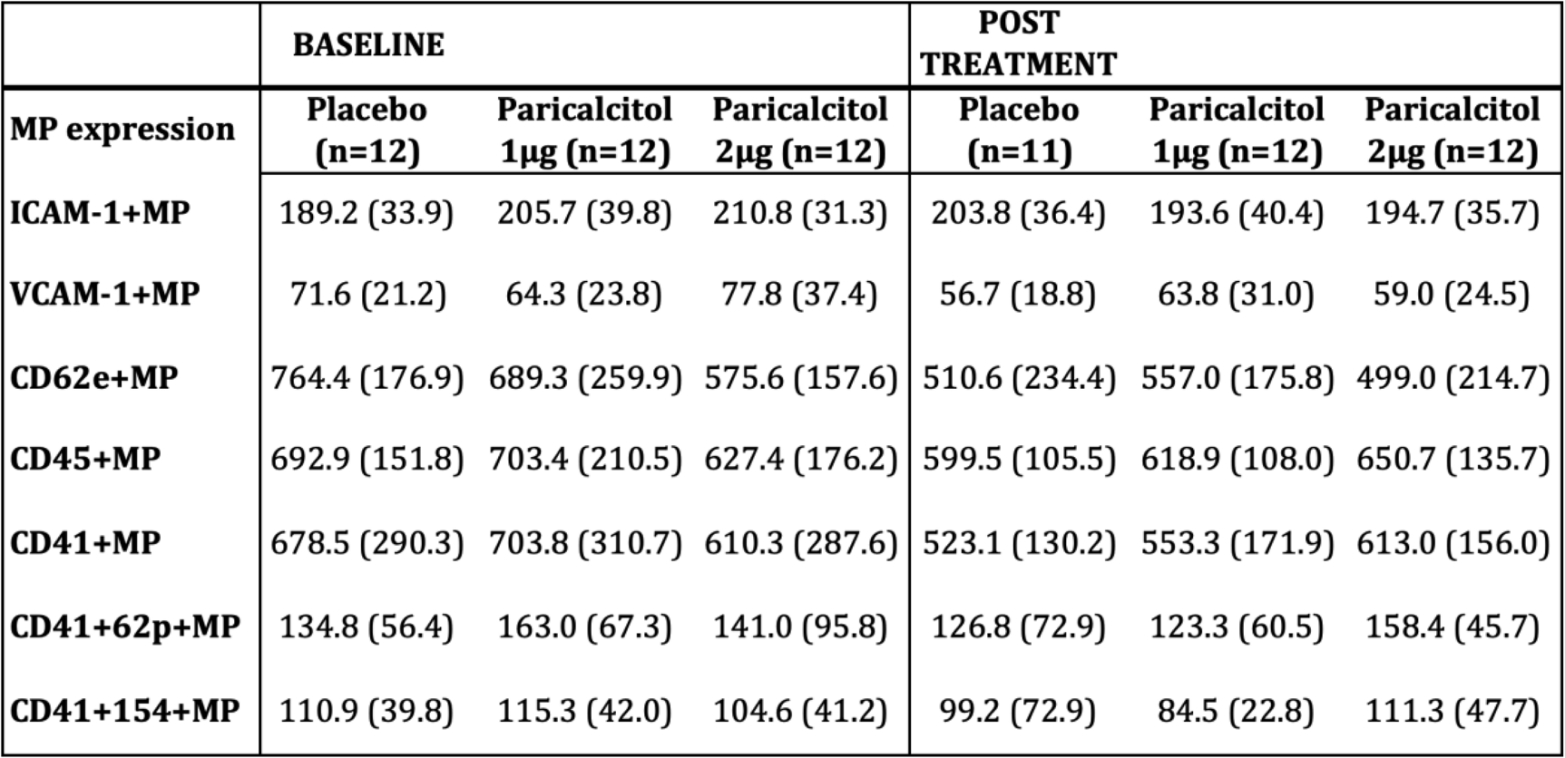
MP concentrations pre- and post-treatment

*ICAM-1* Intercellular adhesion molecule-1, *VCAM-1* Vascular adhesion molecule − 1, *CD62e*^*+*^ Endothelial MPs, *CD45*^*+*^ Leukocyte MPs, *CD41*^*+*^ Platelet MPs, *CD41*^*+*^*62p*^*+*^ Platelet MPs, *CD41*^*+*^ *154*^*+*^ Platelet MPs. Values are expressed as mean (SD)

### Endothelial, platelet and leukocyte microparticles (EMPs, PMPs and LMPs)

**Fig. 4:**
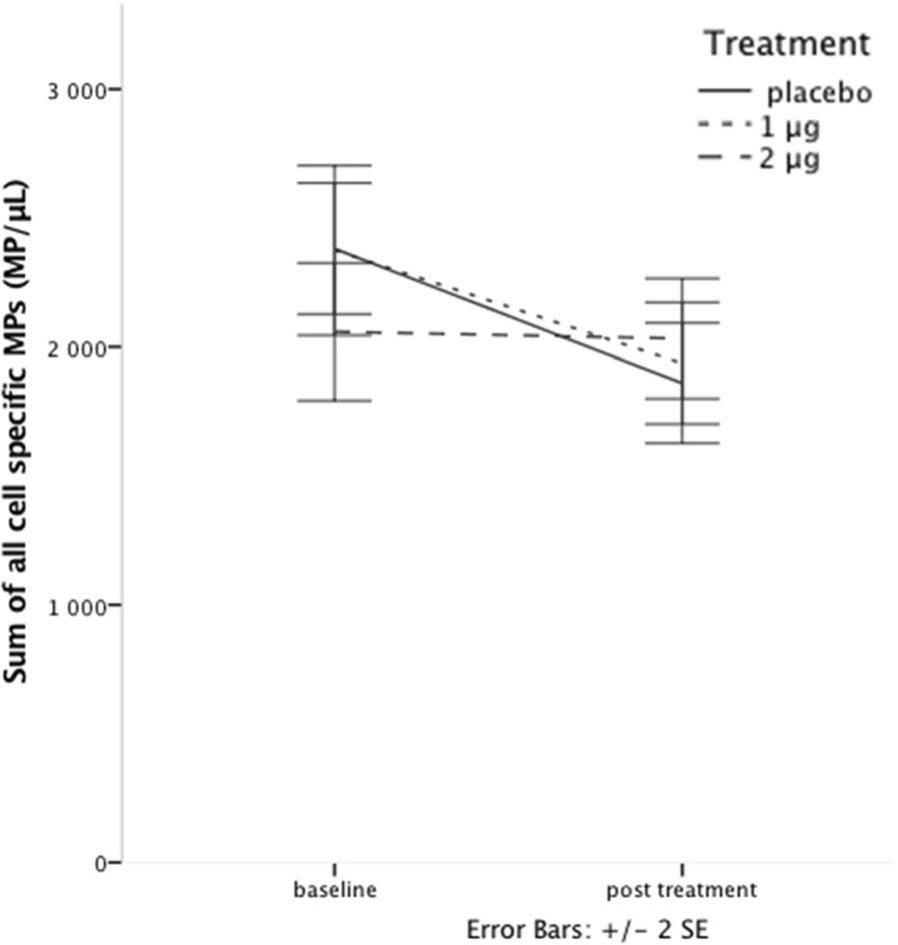
Sum of EMP, PMP and LMP concentrations at baseline and post-treatment show significant decline for placebo and 1 μg, but not for 2 μg of paricalcitol treatment

**Fig. 5:**
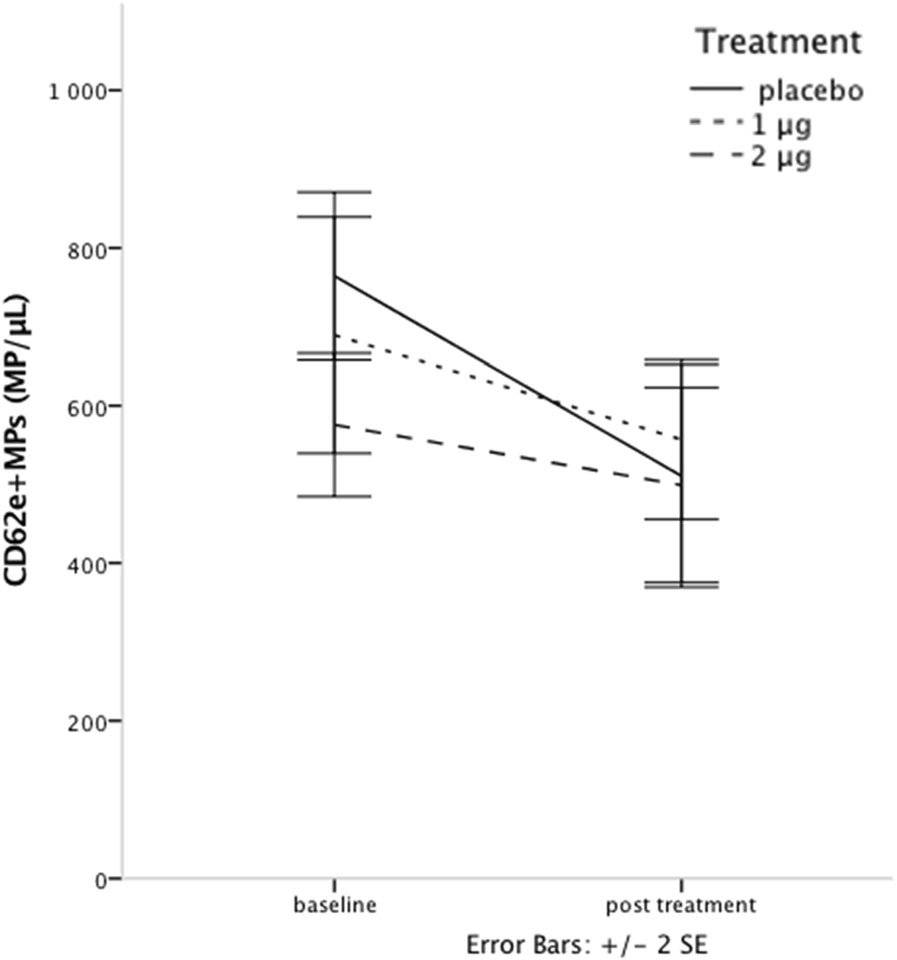
EMP concentrations at baseline and post-treatment show significant decline in the placebo group, but not in paricalcitol treated patients

Concentrations of EMPs, PMPs and LMPs were all well matched between groups at baseline. The repeated measures two-way MANOVA including CD62E^+^ EMPs, CD41^+^ PMPs, CD41^+^ CD62P^+^ PMPs, CD41^+^CD154^+^ PMPs and CD45^+^ LMPs showed a significant decline in MP counts during the study (*p* = 0.001 for the main effect of time), with a non significant interaction between treatment and time (repeated measures MANOVA *p* = 0.08). However, due to the clear pattern seen, further analyses with one-way repeated measures MANOVA were performed. They showed sustained levels in the 2 μg group (*p* = 0.85), and significantly decreasing levels for the 1 μg group (*p* = 0.04), and placebo (*p* = 0.005) (Fig. 4, Table 3). Since our study focused more on the change in EMP levels, further analysis was performed on CD62E^+^ EMPs. There was a significant decrease in CD62E^+^ EMPs across all groups (*p* < 0.001) during the study, as for all MPs. For CD62E^+^ EMPs, this was however due to a significant decline in only the placebo group (paired t-test, *p* = 0.002). The concentrations of CD62E^+^ EMPs in the 1 and 2 μg groups did not decrease significantly during the study (1 μg, paired t-test, *p* = 0.10, and 2 μg, *p* = 0.3), but did change slightly in a dose dependent manner in absolute numbers (Fig. 5, Table 3).

## Discussion

Paricalcitol treatment for 12 weeks to CKD patients in stage 3–4 induces a decline relative to placebo in concentrations of ICAM-1 positive MPs, where soluble ICAM-1 is a marker known to be implicated in atherosclerosis and to correlate with risk of future cardiovascular events [53]. This is in line with our previous findings in the SOLID study, with a maintained endothelial function and reduced inflammation in patients treated with 2 μg of paricalcitol [15, 22]. These findings were also accompanied by sustained concentrations of cell activation induced EMPs and PMPs, as well as levels of LMPs in the 2 μg treatment group, whereas the MP concentrations in the placebo group decreased for all measured cell specific MPs.

It is of importance to know the subtype of MPs investigated to correctly interpret the results, however this is not always clearly stated. For EMPs, Jimenez et al. [35] showed that when apoptosis was induced in endothelial cells in-vitro, activation markers on the endothelial cells and cell activation induced CD62E+ EMPs remained stable, while apoptotic markers and the production of apoptotic EMPs increased rapidly. In patients with acute coronary syndrome, three studies have documented a pattern with high concentrations of apoptotic EMPs [35, 55, 56], probably reflecting acute endothelial cell injury due to ischemia, whereas other MP subtypes were not described.

There are few studies performed that investigate changes in EMPs and their expression by an intervention. Two in-vitro studies [14, 17] on endothelial cell lines show lower levels of CD31+ EMPs after vitamin D treatment likely due to a protective effect against apoptosis. Two clinical studies on atorvastatin treatment in non-CKD patients have shown a rise in CD144^+^ EMPs by treatment [57, 58]. Interestingly, statins are believed to have similar endothelial cell protective effects as vitamin D, reducing oxidative stress and upregulating eNOS [59]. Mobarrez et al. interpreted the statin induced rise in EMPs as a protection against apoptosis, and a more healthy and reactive endothelium. On the other hand, Tehrani et al. [58] have shown an impaired endothelial dependent microcirculatory function in patients with type 1 diabetes during high dose statin treatment, although in that case it might have been due to an impaired glucose control. Augustine et al. [60] reported in a study of apoptotic EMP- and PMP-response to dobutamine stress echocardiography, that patients with signs of coronary disease (wall motion abnormalities) did not react with elevated CD31^+^ EMPs or PMPs, whereas patients with a normal stress-test did. These results seem to indicate, at least in some settings, that a dysfunctional endothelium might be less reactive, not producing more MPs in response to stress or other events, in the same way as in healthy controls or treated patients.

Concentrations of MPs expressing ICAM-1 in our patients were declining in the treatment groups with a significant interaction with treatment. ICAM-1 is an activation marker, implicated in the atherosclerotic process [50] and is believed to correlate to atherosclerotic events [51–53]. Our results are in line with previous findings [21, 61] of decreasing levels of soluble ICAM-1 with vitamin D treatment in patients with CKD, believed to be a sign of a less pro-atherosclerotic endothelium.

We found an overall decline in the levels of cell-specific MP subtypes, however with sustained levels for the 2 μg treatment group. For CD62E^+^ EMPs there were sustained levels in both treatment groups with decreasing levels in placebo treated patients. This finding is especially interesting since we have previously shown, in these patients, that treatment maintained endothelial function measured by FMD [22]. In line with former interventional studies, decreasing levels of activation-induced EMPs may indicate less reactive potential in a chronically dysfunctional endothelium. Vitamin D treatment might then be protective against this ongoing process. However, these findings demand further research.

High levels of CD31^+^ 41^−^ and CD144^+^ EMPs seem to correlate to endothelial dysfunction and to future risk of cardiovascular morbidity and mortality [38–42, 46–49]. It is however unknown how changes over time relate to risk and treatment. Previous results are difficult to directly relate to our findings since only one of these studies investigated CD62^+^ EMPs [46], and since we, in the present study, did not use healthy controls. However, we do have a well characterized population, with sustained endothelial function and decreasing levels of proinflammatory cytokines in the 2 μg treatment group. Our findings with reduced concentrations of ICAM-1 positive MPs and sustained EMP, PMP and LMP concentrations might, in the light of our previous findings, be interpreted as a protective effect of paricalcitol on the vasculature.

The main limitations of the present randomized double blind study is that it is of short duration and with a small number of participants, limiting the generalizability. Despite the randomized design, there were differences in age, and differences in absolute numbers of CV disease in the three groups. Since all outcome measures, as well as measures of kidney function and laboratory parameters, were well matched at baseline, we do not believe that these differences have affected the results. However, we do acknowledge this as a limitation since age and previous CV disease is of great importance in the development of vascular disease. Samples were frozen and thawed for the analyses, however the techniques used should be robust for this issue. MPs are still new as biomarkers and there are very few interventional studies performed, making the results more difficult to interpret. This is to our knowledge the first study reporting on changes in MP concentrations in a randomized interventional trial in CKD patients, and our findings needs further investigations.

## Conclusions

In this study we show that active vitamin D treatment with paricalcitol in CKD patients reduce concentrations of ICAM-1 positive MPs, indicating a less pro-atherosclerotic endothelium. The previously reported maintained endothelial function measured by FMD in the 2 μg treatment group, was associated with maintained concentrations of cell-activation induced EMPs, PMPs and LMPs, which might be interpreted as markers of an endothelium with preserved reactive potential.

## Data Availability

Blinded data is available at request from KL or JS.

## Abbreviations

CKD: Chronic kidney disease
CVD: Cardiovascular disease
eGFR: estimated glomerular filtration rate
FMD: Flow mediated vasodilatation
HD: Hemodialysis
ICAM-1: Intercellular adhesion molecule 1
MDRD: Modification of Diet in Renal Disease
MPs: Microparticles
PPP: Platelet poor plasma
PS: Phosphatidylserine
PTH: Parathyroid hormone
PWV: Pulse wave velocity
VCAM-1: Vascular cell adhesion molecule 1

## References

[1] Levey AS, Coresh J, Balk E, Kausz AT, Levin A, Steffes MW, Hogg RJ, Perrone RD, Lau J, Eknoya G (2003). Clinical guidelines National Kidney Foundation practice guidelines for chronic kidney disease: evaluation, Classificaiton, and stratification. Ann Intern Med.

[2] Piepoli MF, Hoes AW, Agewall S, Albus C, Brotons C, Catapano AL, Cooney MT, Corr U, Cosyns B, Deaton C (2016). 2016 European guidelines on cardiovascular disease prevention in clinical practice. Eur Heart J.

[3] Ronco C, Di Lullo L (2014). Cardiorenal syndrome. Heart Fail Clin.

[4] Stam F, van Guldener C, Becker A, Dekker JM, Heine RJ, Bouter LM, Stehouwer CD (2006). Endothelial dysfunction contributes to renal function-associated cardiovascular mortality in a population with mild renal insufficiency: the Hoorn study. J Am Soc Nephrol.

[5] Bock JS, Gottlieb SS (2010). Cardiorenal syndrome: new perspectives. Circulation.

[6] Himmelfarb J, Stenvinkel P, Ikizler TA, Hakim RM (2002). Perspectives in renal medicine: the elephant in uremia: oxidant stress as a unifying concept of cardiovascular disease in uremia. Kidney Int.

[7] Leonard O, Spaak J, Goldsmith D (2013). Regression of vascular calcification in chronic kidney disease - feasible or fantasy? A review of the clinical evidence. Br J Clin Pharmacol.

[8] Schlieper G, Schurgers L, Brandenburg V, Reutelingsperger C, Floege J (2016). Vascular calcification in chronic kidney disease: an update. Nephrol Dial Transplant.

[9] de Zeeuw D, Agarwal R, Amdahl M, Audhya P, Coyne D, Garimella T, Parving H-H, Pritchett Y, Remuzzi G, Ritz E (2010). Selective vitamin D receptor activation with paricalcitol for reduction of albuminuria in patients with type 2 diabetes (VITAL study): a randomised controlled trial. Lancet.

[10] Kumar V, Yadav AK, Lal A, Kumar V, Singhal M, Billot L, Gupta KL, Banerjee D, Jha V (2017). A randomized trial of vitamin D supplementation on vascular function in CKD. J Am Soc Nephrol.

[11] Levin A, Tang M, Perry T, Zalunardo N, Beaulieu M, Dubland JA, Zerr K (2017). Randomized controlled trial for the effect of vitamin D supplementation on vascular stiffness in CKD. Clin J Am Soc Nephrol.

[12] Thadhani R, Appelbaum E, Pritchett Y, Chang Y, Wenger J, Tamez H, Bhan I, Agarwal R, Zoccali C, Wanner C (2012). Vitamin D therapy and cardiac structure and function in patients with chronic kidney disease: the PRIMO randomized controlled trial. JAMA.

[13] Aranow C (2011). Vitamin D and the immune system. J Investig Med.

[14] Jia X, Xu J, Gu Y, Gu X, Li W, Wang Y (2017). Vitamin D suppresses oxidative stress-induced microparticle release by human umbilical vein endothelial cells. Biol Reprod.

[15] Mansouri L, Lundwall K, Moshfegh A, Jacobson SH, Lundahl J, Spaak J (2017). Vitamin D receptor activation reduces inflammatory cytokines and plasma MicroRNAs in moderate chronic kidney disease – a randomized trial. BMC Nephrol.

[16] Vojinovic J (2014). Vitamin D receptor agonists’ anti-inflammatory properties. Ann N Y Acad Sci.

[17] Xu J, Jia X, Gu Y (2017). Vitamin D reduces oxidative stress – induced Procaspase-3/ROCK1 activation and MP release by placental trophoblasts. J Clin Endocrinol Metab.

[18] De Borst MH, Hajhosseiny R, Tamez H, Wenger J, Thadhani R, Goldsmith DJA (2013). Active vitamin D treatment for reduction of residual proteinuria: a systematic review. J Am Soc Nephrol.

[19] Han T, Rong G, Quan D, Shu Y, Liang Z, She N, Liu M, Yang B, Cheng G, Lv Y (2013). Meta-analysis: the efficacy and safety of paricalcitol for the treatment of secondary hyperparathyroidism and proteinuria in chronic kidney disease. Biomed Res Int.

[20] Xu L, Wan X, Huang Z, Zeng F, Wei G, Fang D, Deng W, Li Y (2013). Impact of vitamin D on chronic kidney diseases in non-dialysis patients: a meta-analysis of randomized controlled trials. PLoS One.

[21] Chitalia N, Ismail T, Tooth L, Boa F, Hampson G, Goldsmith D, Kaski JC, Banerjee D (2014). Impact of vitamin D supplementation on arterial vasomotion, stiffness and endothelial biomarkers in chronic kidney disease patients. PLoS One.

[22] Lundwall K, Jörneskog G, Jacobson SH, Spaak J (2015). Paricalcitol, microvascular and endothelial function in non-diabetic chronic kidney disease: a randomized trial. Am J Nephrol.

[23] Zoccali C, Curatola G, Panuccio V, Tripepi R, Pizzini P, Versace M, Bolignano D, Cutrupi S, Politi R, Tripepi G (2014). Paricalcitol and endothelial function in chronic kidney disease trial. Hypertension.

[24] Lundwall K, Jacobson S, Jörneskog G, Spaak J (2018). Treating endothelial dysfunction with vitamin D in chronic kidney disease: a meta-analysis. BMC Nephrol.

[25] Kendrick J, Andrews E, You Z, Moreau K, Nowak KL, Farmer-Bailey H, Seals DR, Chonchol M (2017). Cholecalciferol, calcitriol, and vascular function in CKD: a randomized, double-blind trial. Clin J Am Soc Nephrol.

[26] Thethi TK, Bajwa MA, Ghanim H, Jo C, Weir M, Gold AB, Umpierrez G, Desouza C, Dandona P, Fang-hollingsworth Y (2015). Effect of paricalcitol on endothelial function and inflammation in type 2 diabetes and chronic kidney disease. J Diabetes Complicat.

[27] Thadhani R, Wenger J, Tamez H, Cannata J, Thompson BT, Andress D, Manning WJ, Solomon SD (2012). Vitamin D therapy and cardiac structure and function in patients with chronic kidney disease. JAMA.

[28] Wang AYM, Fang F, Chan J, Wen YY, Qing S, Chan IHS, Lo G, Lai KN, Lo WK, Lam CWK (2014). Effect of paricalcitol on left ventricular mass and function in CKD-the OPERA trial. J Am Soc Nephrol.

[29] Berezin A, Zulli A, Kerrigan S, Petrovic D, Kruzliak P (2015). Predictive role of circulating endothelial-derived microparticles in cardiovascular diseases. Clin Biochem.

[30] Burger D, Schock S, Thompson CS, Montezano AC, Hakim AM, Touyz RM (2013). Microparticles: biomarkers and beyond. Clin Sci (London, England : 1979).

[31] Markiewicz M, Richard E, Marks N, Ludwicka-Bradley A (2013). Impact of endothelial microparticles on coagulation, inflammation, and angiogenesis in age-related vascular diseases. J Aging Res.

[32] Buendía P, De Oca AM, Madueño JA, Merino A, Martín-Malo A, Aljama P, Ramírez R, Rodríguez M, Carracedo J (2015). Endothelial microparticles mediate inflammation-induced vascular calcification. FASEB J.

[33] Burger D, Turner M, Munkonda MN, Touyz RM (2016). Endothelial microparticle-derived reactive oxygen species: role in endothelial signaling and vascular function. Oxidative Med Cell Longev.

[34] Erdbrügger U, Le TH (2016). Extracellular vesicles in renal diseases: more than novel biomarkers?. J Am Soc Nephrol.

[35] Jimenez JJ, Jy W, Mauro LM, Soderland C, Horstman LL, Ahn YS (2003). Endothelial cells release phenotypically and quantitatively distinct microparticles in activation and apoptosis. Thromb Res.

[36] Abid Hussein MN, Boing AN, Sturk A, Hau CM, Nieuwland R (2007). Inhibition of microparticle release triggers endothelial cell apoptosis and detachment. Thromb Haemost.

[37] Perez-Casal M, Downey C, Cutillas-Moreno B, Zuzel M, Fukudome K, Toh CH (2009). Microparticle-associated endothelial protein C receptor and the induction of cytoprotective and anti-inflammatory effects. Haematologica.

[38] Dursun I (2009). The relationship between circulating endothelial microparticles and arterial stiffness and atherosclerosis in children with chronic kidney disease. Nephrol Dial Transplant.

[39] Amabile N, Guerin AP, Leroyer AP, Mallat Z, Nguyen C, Boddaert J, London GM, Tedgui A, Boulanger CM (2005). Circulating endothelial microparticles are associated with vascular dysfunction in patients with end-stage renal failure. J Am Soc Nephrol.

[40] Esposito K, Ciotola M, Schisano B, Gualdiero R, Sardelli L, Misso L, Giannetti G, Giugliano D (2006). Endothelial microparticles correlate with endothelial dysfunction in obese women. J Clin Endocrinol Metab.

[41] Parker B, Al-Husain A, Pemberton P, Yates AP, Ho P, Gorodkin R, Teh LS, Alexander MY, Bruce IN (2014). Suppression of inflammation reduces endothelial microparticles in active systemic lupus erythematosus. Ann Rheum Dis.

[42] Wang JM, Su C, Wang Y, Huang YJ, Yang Z, Chen L, Wu F, Xu SY, Tao J (2009). Elevated circulating endothelial microparticles and brachial-ankle pulse wave velocity in well-controlled hypertensive patients. J Hum Hypertens.

[43] Faure V (2006). Elevation of circulating endothelial microparticles in patients with chronic renal failure. J Thromb Haemost.

[44] Soriano S, Carmona A, Triviño F, Rodriguez M, Alvarez-Benito M, Martín-Malo A, Alvarez-Lara M-A, Ramírez R, Aljama P, Carracedo J (2014). Endothelial damage and vascular calcification in patients with chronic kidney disease. Am J Physiol Renal Physiol.

[45] Trappenburg MC, Van Schilfgaarde M, Frerichs FCP, Spronk HMH, Ten Cate H, De Fijter CWH, Terpstra WE, Leyte A (2012). Chronic renal failure is accompanied by endothelial activation and a large increase in microparticle numbers with reduced procoagulant capacity. Nephrol Dial Transplant.

[46] Amabile N, Heiss C, Chang V, Angeli FS, Damon L, Rame EJ, McGlothlin D, Grossman W, De Marco T, Yeghiazarians Y (2009). Increased CD62e+ endothelial microparticle levels predict poor outcome in pulmonary hypertension patients. J Heart Lung Transplant.

[47] Amabile N, Guérin AP, Tedgui A, Boulanger CM, London GM (2012). Predictive value of circulating endothelial microparticles for cardiovascular mortality in end-stage renal failure: a pilot study. Nephrol Dial Transplant.

[48] Nozaki T, Sugiyama S, Sugamura K, Ohba K, Matsuzawa Y, Konishi M, Matsubara J, Akiyama E, Sumida H, Matsui K (2010). Prognostic value of endothelial microparticles in patients with heart failure. Eur J Heart Fail.

[49] Sinning JM, Losch J, Walenta K, Böhm M, Nickenig G, Werner N (2011). Circulating CD31 +/Annexin V + microparticles correlate with cardiovascular outcomes. Eur Heart J.

[50] Libby P (2012). Inflammation in atherosclerosis. Arterioscler Thromb Vasc Biol.

[51] Kocijancic M, Cubranic Z, Vujicic B, Racki S, Dvornik S, Zaputovic L (2016). Soluble intracellular adhesion molecule-1 and omentin-1 as potential biomarkers of subclinical atherosclerosis in hemodialysis patients. Int Urol Nephrol.

[52] Papayianni A, Alexopoulos E, Giamalis P, Gionanlis L, Belechri AM, Koukoudis P, Memmos D (2002). Circulating levels of ICAM-1, VCAM-1, and MCP-1 are increased in haemodialysis patients: association with inflammation, dyslipidaemia, and vascular events. Nephrol Dial Transplant.

[53] Stenvinkel P, Lindholm B, Heimburger M, Heimburger O (2000). Elevated serum levels of soluble adhesion molecules predict death in pre-dialysis patients: association with malnutrition, inflammation, and cardiovascular disease. Nephrol Dial Transplant.

[54] Bevc S, Sabic S, Hojs R (2008). Atherosclerosis in hemodialysis patients--the role of microinflammation. Ren Fail.

[55] Jung C, Sorensson P, Saleh N, Arheden H, Ryden L, Pernow J (2012). Circulating endothelial and platelet derived microparticles reflect the size of myocardium at risk in patients with ST-elevation myocardial infarction. Atherosclerosis.

[56] Ye S, Shan X-F, Han W-Q, Zhang Q-R, Gao J, Jin A-P, Wang Y, Sun C-F, Zhang S-L (2017). Microparticles from patients undergoing percutaneous coronary intervention impair vasodilatation by uncoupling endothelial nitric oxide synthase. Shock.

[57] Mobarrez F, Egberg N, Antovic J, Bröijersen A, Jörneskog G, Wallén H (2012). Release of endothelial microparticles in vivo during atorvastatin treatment; a randomized double-blind placebo-controlled study. Thromb Res.

[58] Tehrani S, Mobarrez F, Lins P-E, Adamson U, Wallén HN, Jörneskog G (2013). Impaired endothelium-dependent skin microvascular function during high-dose atorvastatin treatment in patients with type 1 diabetes. Diab Vasc Dis Res.

[59] Steven S, Münzel T, Daiber A (2015). Exploiting the pleiotropic antioxidant effects of established drugs in cardiovascular disease. Int J Mol Sci.

[60] Augustine D, Ayers LV, Lima E, Newton L, Lewandowski AJ, Davis EF, Ferry B, Leeson P (2014). Dynamic release and clearance of circulating microparticles during cardiac stress. CircRes.

[61] Naeini A, Moeinzadeh F, Vahdat S, Ahmadi A, Hedayati Z, Shahzeidi S (2017). The effect of vitamin D administration on intracellular adhesion molecule-1 and vascular cell adhesion molecule-1 levels in hemodialysis patients: a placebo-controlled, double-blinded clinical trial. J Res Pharm Pract.

